# *Legionella* spp. in a Dental Office—Current State of Knowledge

**DOI:** 10.3390/pathogens14060512

**Published:** 2025-05-22

**Authors:** Jolanta Szymańska

**Affiliations:** Department of Comprehensive Paediatric and Adult Dentistry, Medical University of Lublin, 20-059 Lublin, Poland; jolanta.szymanska@umlub.pl

**Keywords:** *Legionella pneumophila*, dentistry, dental unit waterlines, DUWL, bioaerosol, biological hazards, occupational hazards

## Abstract

Conditions in dental offices are conducive to *Legionella pneumophila* infections. This is mainly related to the use of a dental unit in the daily clinical work, which is the basic equipment of the office. Water discharged from the dental unit waterlines (DUWLs) and the working tips of the dental unit generates splatter/spatter and bioaerosol, constituting the main sources of potential infection and posing a health threat to both patients and professional dental staff. This article presents a narrative review on the presence and risk associated with *Legionella* spp., particularly the species *L. pneumophila*, in the dental office. This paper summarizes current knowledge and offers readers practical references, especially useful in everyday clinical dental practice.

## 1. Introduction

Conditions in dental offices are conducive to *Legionella pneumophila* infections, posing a health threat to patients and professional staff. This is mainly related to the use of a dental unit in the daily clinical work, which is the basic equipment of the office. Due to the need to cool down rotary equipment and processed tissues, as well as to rinse the mouth during preventive and therapeutic procedures, the water flowing from the working tips is necessary in dental units [1]. In the case of microbiological contamination of the water in the Dental Unit Waterline (DUWL), with the presence of *L. pneumophila* bacteria, the water becomes a potential source of infection, especially since the working tips of the unit connected to the DUWL contribute to the formation of spray and aerosol [2,3,4]. It is noted that, in particular, elderly patients and those with weakened immunity are at risk of serious respiratory infections if the water contains pathogens such as *L. pneumophila* and *Pseudomonas* spp. [5].

The aim of this narrative review on the presence and risk associated with *Legionella* spp., particularly *L. pneumophila*, in the dental office was to provide an updated synthesis of the available literature. The author, without the involvement of other individuals or the use of artificial intelligence, conducted a literature review from the year 2020, focusing on the topics of *Legionella* spp., *L. pneumophila*, dentistry, dental unit waterlines, biological hazards, and occupational hazards. A key inclusion criterion was the relevance of the most recent findings from observational, descriptive, analytic studies, and systematic reviews.

## 2. Routes of Infection with *L. pneumophila* in Dental Practice

The working tips of the dental unit—high-speed handpiece, low-speed handpiece, air–water syringe, and ultrasonic scaler—are the main sources of aerosols in a dental office [6]. Smaller aerosol components mainly contaminate the air in the treatment room, while larger ones are responsible for contaminating various surfaces. Microbiological contamination generated by high-speed handpieces is significantly higher than that from low-speed handpieces [4].

Two main routes of *L. pneumophila* transmission in a dental office should be distinguished: waterborne droplet route, a droplet water aerosol emitted by the dental unit’s working tips, which may contain *Legionella* spp. bacteria present in tap water and/or in water from the unit’s reservoir and/or developing in the biofilm on the inner surfaces of the DUWL, as well as saliva-derived droplet route via aerosol emitted by an infected patient. This latter aerosol includes, among other things, saliva, gingival crevicular fluid, tooth tissues, fragments of dental fillings, and dental plaque [7]. According to Rafiee et al., patients’ saliva and nasal fluids should not be considered a significant source of bioaerosols in dentistry. Research shows that a median of 80.15% of operator exposure was attributable to sources other than the patients’ salivary or nasal fluids. Median operators’ exposure from patients’ fluids ranged from 1.45% to 2.75%, and microbiota showed more patients’ nasal bioaerosols than oral bioaerosols [8]. As calculated in earlier studies, a dentist and dental assistant may inhale between 0.014 and 0.12 μL of patient saliva in aerosol form during a 15 min peak exposure period while the dental unit’s working tips are in operation [9].

Fluids released under pressure from the working tips contribute to an increased risk of contamination concentrated within a 30 cm radius, with the overall range of microbiological contamination extending up to 60 cm. The highest level of aerosol splatter points is observed at a maximum working tip speed of 40,000 rpm, while the lowest is recorded at a speed of 10,000 rpm. The highest level of contamination occurs at the operator’s and assistant’s positions. Both the rotational speed and the volume of water released influence the degree of contamination [10]. As early as the mid-1990s, it was shown that during working hours, the average bacterial load in dental office air increases more than threefold. The level of aerobic bacterial load was 1.5 times higher, and anaerobic bacterial load was twice as high compared to the initial baseline level [11]. The aerosol generated under pressure is further set in motion by rotating and vibrating instruments, such as dental drills and scaler tips. As a result, it mixes with the room air and leads to microbiological contamination of the dental office environment [12].

Levels of dental aerosol contamination decrease with distance from its source, as well as over time after the dental procedures are completed. However, studies have not shown a consistent period of time in which contamination levels return to pre-procedure levels [13].

An additional factor that may contribute to contamination by *Legionella* spp. bacteria in the DUWL environment is the backflow of fluids from the oral cavity of an infected patient into the unit’s waterlines. This indirectly becomes a source of bioaerosol contamination. This occurs when the unit’s backflow prevention system does not function properly [14].

When assessing the risk of infection due to exposure to *L. pneumophila* through the water-airborne route in a dental office, four key factors should be considered: the portal of entry, the host’s susceptibility to infection, the virulence of the pathogen, the amount of infectious material, and the mode of transmission [15]. The portal of entry for *L. pneumophila* is the nose and mouth, with the source of infection being an aerosol potentially contaminated with *L. pneumophila*, which contains infectious material with a specific level of virulence. Theoretically, the potential health hazard of *Legionella* to humans is associated with cell concentrations above 10^4^ to 10^5^ CFU/L of water [16]. Additionally, when released under pressure, the aerosol may contribute to more intense inhalation, effectively leading to the inhalation of bioaerosol due to the proximity of the portal of entry and the source of infection. Especially bacteria such as *Mycobacterium tuberculosis* (tuberculosis bacilli) and *L. pneumophila* serogroup 1, transmitted through aerosols, can lead to respiratory diseases such as tuberculosis and Legionnaires’ disease [17,18,19]. As previously mentioned, the immunity—or lack thereof—of the host organism, including the patient and members of the dental team, plays a significant role in susceptibility to infection [10].

## 3. Characteristics of *Legionella pneumophila*

*L. pneumophila* is one of the bacterial species belonging to the genus *Legionella*. It is an aerobic, Gram-negative bacillus found in both natural and artificial aquatic environments, existing in a planktonic form as well as a component of biofilm. *L. pneumophila* is a species of γ-proteobacteria that parasitizes free-living freshwater amoebae, which play a crucial role in the growth and spread of the bacteria [1,20]. *L. pneumophila* multiplies at temperatures between 25 and 37 °C, and its survival temperature range is from 0 to 63 °C. Key nutritional components for the bacteria are amino acids (L-cysteine) and iron. *L. pneumophila* is sensitive to drying, ozone, chlorine, and ultraviolet (UV) radiation. Infection with *L. pneumophila* occurs when contaminated water aerosol is inhaled or when aspiration occurs due to choking, leading to the bacteria entering the respiratory system. The incubation period is 2–10 days. Most cases of the disease caused in humans are triggered by *L. pneumophila* serogroup 1, one of the 15 serogroups of this species [1]. Among all *L. pneumophila* species, serogroup 1 is responsible for at least 70% of all cases of legionellosis in the United States and Europe, making it the most clinically significant [21]. In the case of *Legionella* spp., attention is drawn to the importance of biofilm presence, which plays a significant role in chronic colonization by microorganisms, including in water distribution systems and plumbing installations that are not properly monitored microbiologically [21]. It has already been shown that changes in temperature and precipitation related to climate warming may impact the increase in the incidence of legionellosis in Europe [22,23]. The disease can manifest as one of two clinically distinct syndromes: Legionnaires’ disease, which has a severe course, and Pontiac fever, a flu-like form with a mild course. Molecular mechanisms enabling intracellular parasitism and pathogenicity of *L. pneumophila* were described in detail by Canadian researchers and published at the beginning of 2024 [24].

## 4. The Prevalence of *Legionella* spp. in DUWLs and Dental Office Environments

The results from studies published in recent years on the presence of *Legionella* spp. and the species *L. pneumophila* indicate that their prevalence in DUWLs varies widely. Microbiological contamination assessed using the plate method in 20 Indian dental clinics, focusing on the presence of *Aspergillus*, *Acinetobacter*, *Pseudomonas aeruginosa*, and *Legionella*, confirmed that *Legionella* was the most frequently detected microorganism, found in 70% of water samples from air–water syringes and in 50% from scalers and air rotor handpieces. For comparison, the prevalence of other bacteria and fungi was as follows: *Pseudomonas aeruginosa* and *Acinetobacter*–10% (in water from scalers and air rotor handpieces), and *Aspergillus*–10% in water from air–water syringes [25]. In a German study conducted in 2019 and 2020, during which 3789 water samples were collected from 459 dental units, the presence of *Legionella* spp. was detected in 36.4% of the samples. The dominant species was *Legionella anisa*, accounting for 97.89% of the positive cases. Identification was carried out using biotyping with MALDI-TOF (Matrix-Assisted Laser Desorption and Ionization–Time-of-Flight Mass Spectrometry). The prevalence rates were as follows: *L. anisa*–95.28%, a combination of *L. anisa* and *L. pneumophila*–2.61%, and *L. pneumophila* alone–2.11%. The occurrence of the latter species was relatively rare [26].

In eight European countries, the following prevalence rates of *Legionella* spp. were reported: 9% in Denmark and Spain, 0% in the United Kingdom, the Netherlands, Greece, Germany, and Ireland, and 58% and 33.3% in Italy. In four Middle Eastern countries (Turkey, Iraq, Iran, and Jordan), the overall average prevalence of *L. pneumophila* was 23.5%, with individual rates ranging from 0% to 86.7%. Previous studies from Italy and South Africa, as cited by Khajezadeh et al., reported the prevalence of *Legionella* spp. in DUWL water samples at rates of 33.3% and 33%, respectively [27].

Bayani et al., in a study published in 2023 based on a systematic review and meta-analysis of research published between 1976 and 2020, estimated the prevalence of *L. pneumophila* and *P. aeruginosa* in DUWLs to be 12.0% and 8.0%, respectively [8]. Studies on the microbial load and microbiome in the water of 226 dental units in the Netherlands, as well as factors influencing these parameters, revealed that after the so-called biofilm release following overnight stagnation, *Legionella* spp., amoebae, and fungi were detected in 71%, 43%, and 98% of all units, respectively. Moreover, more *Legionella* spp. bacteria were present in the DUWLs connected directly to the potable water supply than in those connected to an external reservoir integrated into the dental unit’s structure [28]. An analysis of data from articles published between 2000 and the end of July 2016, containing information on the prevalence of *Legionella* spp. in water sources in Iran, confirmed a high occurrence in dental facilities, with a prevalence rate of 23.6%. The most frequently detected species was *L. pneumophila*, with a prevalence of 60.5% [29]. In 30 Italian dental offices where water samples were collected from both taps and DUWLs, the overall prevalence of *L. pneumophila* (from both sources) was 78.33%, while in samples taken exclusively from DUWLs, the prevalence increased to 86.67%. The presence of amoebae belonging to the species *Vermamoeba (Hartmannella) vermiformis* was also confirmed in 60% of water samples taken from the handpiece outlets of dental units [30].

## 5. DUWL Biofilm and *Legionella* spp.

The presence of biofilm on the inner walls of the tubing forming the DUWL is a significant source of microbiological contamination of water in dental units, including by opportunistic pathogens [10,31]. Factors conducive to DUWL biofilm formation have been described in detail in previous publications [8,12,32]. Aquatic biofilms attached to water-bearing surfaces in dental equipment can amplify the number of bacteria and other microorganisms present in water used for dental treatment, including potentially pathogenic microorganisms such as *P. aeruginosa*, nontuberculous mycobacteria (NTM) and *Legionella* species [33].

According to some researchers, *P. aeruginosa* and *L. pneumophila* are the most common opportunistic pathogens found in DUWL biofilms, raising concerns about cross-infections, particularly in immunocompromised individuals [34,35]. The formation of biofilm containing *L. pneumophila* is influenced by other microorganisms present in the biofilm, the presence of collagen-like adhesins, amoebae, flagella, iron ions, and signaling molecules that regulate various important biological processes in bacteria, such as cyclic diguanylate (c-di-GMP) and quorum sensing. The biofilm environment provides favorable conditions for bacterial proliferation and protection against chemical and physical factors, including those used for DUWL disinfection. It also contributes to the increased virulence of *Legionella* bacilli. Biofilm is also a source from which *L. pneumophila* bacilli spread by transitioning into planktonic forms [36].

The role of biofilm in the survival and re-colonization of *L. pneumophila* in water distribution systems is confirmed by research findings. The disinfection strategy using sodium chloride (at various concentrations) was effective against planktonic *L. pneumophila* cells but not against biofilms containing these bacteria. Additionally, this effect was less pronounced with the age of the biofilm; older biofilms were more persistent than younger ones [7].

DUWL can also be an important site for replication of free-living amoebae and protozoa, which facilitate the maintenance of intracellular pathogenic bacteria, thereby increasing their resistance to disinfectants. It has been confirmed that there is evidence that amoebae accumulate around bacterial biofilms, and their numbers are even 300 times higher in the outlet water, flowing from DUWL, compared to tap water, which is one of the possible water sources for dental units. Undoubtedly, this can affect the presence of *L. pneumophila* both in the biofilm itself and in the water flowing from the DUWL [37]. Amoeba are known to be more resistant to antimicrobial agents than bacteria and feed on both live and dead biofilms [38]. According to Hoogenkamp et al., biofilm removal, rather than killing, is of prime importance as no significant difference in the prevalence of amoeba was found between the different types of disinfectant applied [28].

It is estimated that approximately 95% of all microbial cells present in water distribution systems exist in the form of biofilm on the surface of the pipes, while only 5% are found in the water phase. Studies by Zayed et al. on the occurrence of *Legionella* bacilli in DUWLs indicate that they were present with significantly lower frequency in water compared to biofilms. This confirms the importance of using disinfection methods primarily targeted at biofilms in DUWL tubing [7,39].

Considering the interdependence between the biofilm microbiome and the water environment of DUWLs [31], preventing biofilm formation in DUWLs means effectively preventing microbiological contamination in dental practice. It has been found that water disinfection using a method based on osmosis and chlorine dioxide, especially when integrated with an additional water desalination procedure before entering the dental units, prevents the infiltration of inorganic and organic compounds that could promote biofilm formation in the waterlines [40].

To sum up, it should be stated that infection control guidelines should focus on the presence and control of biofilm formation as this is the source of reinfection of the effluent water from DUWLs.

## 6. Water from DUWLs and *Legionella* spp.

Patients and dental staff are exposed to water from DUWLs in three ways: through splashing onto the skin and mucous membranes, aerosols, and possible ingestion of water during dental care. The latter two, as mentioned in the introductory part of the article, are particularly significant in terms of the risk of infection with *Legionella* spp.

Currently, there are no official norms or regulations regarding the microbiological quality of DUWL water that would apply in dental offices and could be enforced by relevant services. Current standard bacterial contamination levels are recommendations that recommend not exceeding a certain level of microbiological load in DUWL water.

In the absence of any applicable standards for the quality of water in dental offices, it is generally accepted that it must at least meet the criteria of drinking water. Recommendations regarding water standards used in dental offices were established years ago. The American Centers for Disease Control and Prevention (CDC) recommends that the number of heterotrophic microorganisms (HPC–heterotrophic plate count) in dental unit water should not exceed 500 CFU/mL for non-surgical dental procedures [41]. In the EU, there are currently no specific guidelines regarding water quality in DUWLs. In some countries, the reference point is the standard for drinking water established by the European Commission, which sets the limit for heterotrophic bacterial load at 100 CFU/mL [42]. This means an aerobic colony count of <100 CFU/mL after 72 h of incubation at 22 °C or 20 CFU/mL after 24 h of incubation at 37 °C [43].

The recommendations focus on the overall microbiological load of water in DUWLs. However, they overlook the critically important fact, confirmed in studies, of the presence of opportunistic pathogenic bacteria in DUWL water, such as *P. aeruginosa*, *L. pneumophila*, and nontuberculous *Mycobacterium* [44,45]. All the more so because, according to the World Health Organization (WHO) water quality standards, certain microorganisms such as *Acinetobacter*, *Aspergillus*, *P. aeruginosa*, and *L. pneumophila* should not be present in hospitals and healthcare facilities, as they can lead to opportunistic infections in immunocompromised patients [46].

Therefore, there is a necessity for stringent quality control and certification of DUWL water, with particular emphasis on ensuring the absence of the mentioned pathogens due to their potential risk to human health [47].

It has been recognized that the factors which may influence the presence of *Legionella* spp. in the water of dental unit waterlines (DUWLs) are as follows: the water source from which it is supplied to the unit, the overall microbiological load of the water present in the DUWL, which is affected by the composition of the biofilm, the water temperature in the DUWL and in the dental office room, the backflow of patient saliva into the DUWL, the type of procedures and disinfectants used for DUWL maintenance, as well as the model(s) of the dental unit [14]. For example, water temperatures in dental units above 20 °C contribute to increased *Legionella* colonization, while the risk of *Legionella* contamination is 28.8% lower at temperatures below 20 °C. The KAVO 1058 unit model (KaVo Dental) is characterized by significantly lower *Legionella* colonization compared to the Sirona C5+ and C8 models (Dentsply Sirona) [26]. The presence of *Legionella* bacteria in the biofilm of inadequately cleaned dentures or poor oral hygiene in patients of the dental office—individuals infected with these bacteria—constitutes a source of contamination for DUWLs [48].

Lizzadro et al. emphasize that the storage tank, the proper functioning of anti-backflow valves, and the disinfection procedures carried out are the main critical points in dental unit management (maintenance/sanitation). They confirm that these factors are crucial for ensuring the safety of both patients and operators, highlighting that there is often a lack of full awareness regarding the real risks involved [14].

Previous data from 2016 in eastern France showed that 91.0% of dental units were supplied with tap water (65.0% were directly supplied with water from the municipal water network, 19.7% with filtered water from the municipal network, and 6.3% with water from the municipal network subjected to osmosis), and one-third (33.6%) of DUWLs had an independent water tank [44]. For example, in Germany, the source of water for dental practices is local municipal water that meets drinking water standards in accordance with the German Drinking Water Regulation [26]. A little more than one-half of the American respondents working in dental offices (51%) indicated that practice in which they worked used distilled water. Other commonly reported water sources were municipal water/tap water (32%), deionized water (19%), commercial or developed bottled water (18%), in-office filtration system (22%), and building filtration system (9%) [49].

As indicated by the systematic review and meta-analysis, different water sources used to supply dental units can have varying impacts on the microbiological contamination of water in DUWLs. On one hand, using tap water resulted in a lower rate of microbiological contamination in the water exiting the unit, while on the other hand, purified water contributed to better microbiological quality of the water exiting the unit, or no significant impact was observed between the type of water source and the microbiological quality of the water exiting the working tips of the unit [50].

It appears that, as previously stated, the ADA standard of 200 CFU/mL was achieved using a closed water system and distilled water treated with a disinfectant, provided it was used, as confirmed in much earlier studies by Kettering et al., which involved the Bio2000 preparation [51]. A closed water system of a dental unit is a system where water is drawn from a reservoir (tank) that belongs to the unit. It typically contains distilled water, to which a selected disinfectant is added. The solution is traditionally prepared by the assisting staff, or the disinfectant is automatically dispensed. According to CDC guidelines regarding distilled water, its microbiological quality should be tested every 6 months [41].

It should be kept in mind that *Legionella* is a part of the community complex. The most prevalent species were pneumophila serogroup 1 (78.53%), followed by *L. anisa* (54.45%) and *L. rubrilucens* (21.99%) which were sometimes present together. The simultaneous presence of different *Legionella* species can result in increased resistance to high temperatures and disinfection, leading to changes in the contamination levels and species diversity [52]. According to Girolamini et al., *Legionella* surveillance must consider bacterial concentration and identification of communities that are influenced by interactions with the environment, water characteristics, and the choice of pipeline system [52].

In summary, it should be stated that there is a need to use water of high microbiological quality in DUWLs by regularly monitoring water sources and maintaining appropriate microbiological quality of the water in the DUWL, installing filters in water tanks, and preventing backflow. This is the most effective way of preventing colonization by pathogens including *Legionella* spp. and the formation of a biofilm in DUWLs with its presence.

Research should be continued in order to develop a consensus on acceptable levels of the total number of microorganisms in water used for dental treatment, complemented by acceptable levels of *nontuberculous mycobacteria* (NTM), especially *Legionella* and amoebae in DUWLs [33].

## 7. The Air in the Dental Office and *Legionella* spp.

The operation of all working tips of the dental unit—especially the ultrasonic scaler, which is the device that contributes most significantly to microbiological air contamination—leads to a substantial increase in the number of bacteria, viruses, and fungi. These microorganisms remain suspended in the air for a prolonged period, posing a risk of infection to both dental staff and patients. Bacteria can spread up to a distance of 1.5 m, while smaller microorganisms, such as viruses, can remain airborne and reach up to 2 m from the patient’s mouth [53]. In the study by Malmgren et al., it was found that among the evaluated dental unit working tips, the air–water syringe generated the highest number of aerosols. It dispersed infectious aerosols containing viruses throughout the entire room, whereas other instruments did so only at close range [54].

As noted, the use of low-volume evacuation (LVE) and high-volume evacuation (HVE) systems effectively reduces aerosol contamination from the ultrasonic scaler, with no significant difference between the different types of suction devices [53]. The number of microorganisms (CFU) released into the air of the dental office during procedures is significantly reduced when a high-volume evacuation (HVE) system is used [55]. HVE systems characterize with the large diameter tubes and larger internal diameters offer less resistance to airflow, so the vacuum flow rate can be higher. Additionally, the tip of the high-volume evacuator can be placed close to the location where aerosol is generated [56]. As demonstrated, the use of an extraoral HVC during simulated dental treatment with a high-speed handpiece reduced splatter (droplet dispersion) at the level of the dentist’s eyes as well as at the level of the simulated patient’s mouth. When the extraoral suction device was used at distances of 10 and 4 inches from the simulated patient’s mouth, less intense splatter was detected [57].

Assuming that the concentration of *L. pneumophila* in DUWLs is the dominant risk factor for infection with the pathogen, Hamilton et al. indicate that increasing the frequency of air exchanges in the treatment room per hour and the use of N95 respirator masks significantly reduces this risk. Increasing air change rates in the treatment room from 1.2 to 10 would achieve an ∼85% reduction in the risk of *L. pneumophila* infection. Additionally, and importantly for the staff, using an N95 respirator dust mask would reduce the risk by approximately 95% [58].

An important element in maintaining air quality in the dental office is the installation of an air conditioner that also functions as an air cleaner (AC). It should be appropriately positioned in relation to the aerosol source and the dentist, and ensure a high airflow through the device, which allows for a reduction in the concentration of bioaerosol components, including microorganisms. This is especially important given the lack of personal protective equipment for the patient [59].

It has been confirmed that DUWL water disinfection based on osmosis and chlorine dioxide (ClO_2_) chlorination has proven effective for both water and air. Air samples were found to be free from the studied *L. pneumophila* and *P. aeruginosa*, while the chlorine content increased from 0 to 0.06 mg/L. Thus, the presence of residual chlorine dioxide activity also enabled a reduction in bacteria in the air, at least up to one meter away from the aerosol source [40].

It should be emphasized that maintaining high microbiological air quality in the treatment room is a crucial factor in preventing the risk of infection, including with *Legionella* bacteria.

## 8. Tests for the Presence of *Legionella* spp.

The presence of *Legionella* bacteria in DUWL water samples and biofilm can be detected using two main types of analyses: cultivation-dependent analysis (CDA) and cultivation-independent analysis (CIA). The independent methods target *Legionella* from the genus to the species level using molecular techniques, including conventional PCR and 16S rRNA sequencing. The use of molecular methods, including those aimed at managing *Legionella*, can significantly enhance the ability to detect, monitor, and control the presence of *Legionella* in water systems, including biofilms—an observation that has been confirmed by studies in recent years. There is also mention of incorporating molecular methods into the monitoring of so-called critical points within the dental office infrastructure, including the dental unit itself [39]. The usefulness of quantitative polymerase chain reaction (qPCR) is also emphasized as a valuable tool for monitoring the presence of opportunistic pathogens [28].

For objective detection, microbiological testing should be conducted by separately collecting water samples from handpiece outlets, the cup filler, and the water bottle or tap water, while also including the suction system. This approach helps to better understand the source of microbial contamination and to ensure that all components are connected to the main disinfection system. It is recommended that these procedures be performed at least every six months [60]. Since the level of *Legionella* spp. contamination determined by culture methods does not reflect the true extent of bacterial abundance, it is recommended to conduct tests for the presence of aerobic heterotrophic bacteria, based on the assumption that *Legionella* spp. are components of the biofilm [61]. Additionally, higher levels of *Legionella* and amoeba contamination in DUWL water may be influenced by environmental conditions that favor their growth, such as elevated temperatures occurring seasonally or specific to a given region [28]. *Legionella* surveillance must consider bacterial concentration and the identification of communities that are influenced by interactions with the environment, water characteristics, and the choice of pipeline system [52].

The R2A agar culture method is a medium used for heterotrophic plate counts in treated potable water and is still applied in monitoring water quality in DUWLs. However, *Legionella* species and nontuberculosis mycobacteria (NTM) are typically not recoverable using R2A media under standard incubation times. It is suggested that the use of flow cytometry (FCM) could serve as a complementary tool, providing more accurate and real-time information immediately after water sample collection, and has the potential to become an essential method for monitoring dental water quality [33].

The results of the validation of a loop-mediated isothermal amplification-based kit for the detection of *L. pneumophila* in environmental samples, conducted by Italian researchers according to ISO/TS 12869:2012, showed that loop-mediated isothermal amplification (LAMP) offers several advantages compared to PCR and real-time PCR: higher sensitivity, specificity, greater amplification efficiency, and higher yields of amplification products than PCR. The analysis time is shorter, and the procedure is simpler. As emphasized by the researchers, this method represents a promising screening tool that should be used in addition to cultural analysis [3].

The research results encourage the implementation of MALDI–TOF MS (mass spectrometry) in routine diagnostics and environmental *Legionella* surveillance, as it displays a reliable and faster identification at the species level [62].

Özmen et al. recommend the dip slide technique method (DSM) as a sensitive and highly practical approach, which can detect and count bacteria more sensitively than conventional methods, such as the surface smear method (SSM), in dental water systems. One of its key advantages is that it does not require experienced personnel or specialized equipment. In their study published last year, they provide a detailed description of the testing procedure [63].

According to the research, the UltraSnap™ ATP device with a Hygienia bioluminometer was used as a useful tool for assessing the microbial load in the dental chair waterlines. The unique design of the test and the swab for sampling allows for quick and easy collection of the swab. This is a complete test for monitoring the level of cleanliness/ATP and the effectiveness of cleaning and disinfection, and the bioluminometer is the fastest device for microbiological assessment of the swab in a few seconds [64].

With the aim of preventing cross-infections associated with the use of dental units and ensuring appropriate microbiological water quality in dental clinics, regular assessments should be conducted using analytical techniques, such as flow cytometry and modern immunological methods.

## 9. Prevention of Cross-Infections in the Dental Office, Including *L. pneumophila*—Selected Aspects

### 9.1. Dental Office Room

In order to properly prevent cross-infections in the dental office environment, which may result from varying degrees of microbiological contamination, including *Legionella* spp., of instruments and devices, furnishings, and air, it is recommended to apply sterilization, appropriately selected disinfectants, as well as ozonation, ionization, ventilation, fogging, and UV light [8].

### 9.2. DUWL Environment

According to Lizzadro et al., the critical points in monitoring the microbiological quality of the DUWL environment are as follows: the microbiological condition of the water storage tank, the lack of backflow prevention valves, and the quality of the disinfection procedures performed. Maintenance and disinfection, often neglected or improperly carried out by those responsible, are frequently underestimated in terms of the actual infection risk to patients and dental office staff [14].

A meta-analysis on the occurrence of general bacterial contamination in DUWLs, published in 2023, indicates that it is very high. According to the ADA, CDC and C-100 standards, the prevalence of bacterial contamination was estimated to be 85.0% (95% confidence interval (CI): 66.0–94.0%), 77.0% (95%CI: 66.0–85.0%), and 69.0% (95%CI: 67.0–71.0%), respectively [8].

As a result of inadequate monitoring, water systems in dental units often contain commonly occurring waterborne microorganisms, including potentially pathogenic ones such as NTM, *P. aeruginosa*, *Legionella*, and those associated with oral diseases. Additionally, the bacterial count significantly exceeds the levels recommended by the CDC, posing a threat to human health [47].

The presence and persistence of microbiological contamination in DUWLs, including varying levels of *L. pneumophila* as previously mentioned, are also related to the need for strict adherence to operational guidelines and the selection of effective disinfectants for DUWLs. Guidelines for maintaining water quality are widely available and should form the basis for developing and approving DUWL monitoring protocols in dental clinics. It is essential that the staff be aware of the risks associated with the water used in practice, undergo regular training, and adhere to the established procedures. According to the recommendations, periodic monitoring of dental units should be conducted in accordance with the manufacturer’s instructions [41,44,65].

### 9.3. The Role of Dental Team

According to Vinh et al., the dental team, armed with up-to-date knowledge on this topic, should implement best practices with full commitment and appropriately respond to elevated levels of microbiological contamination. However, despite the recognition that controlling contamination in DUWLs is crucial, significant gaps in knowledge and daily practices are still observed [49]. According to Dahlen, the identified anomalies in the unacceptable water standards in DUWLs, despite ensuring the appropriate technical conditions of dental units, occur due to neglect or improper water cleaning procedures. This is a result of the ability to follow instructions, rather than the procedure itself [5].

According to European studies on the attitudes of general dentists towards microbiological hazards associated with DUWLs, the majority did not clean, disinfect, or assess the microbiological load in DUWLs [64].

An important issue regarding dental staff is their resistance to infections. Advances in computer-aided vaccine design create the possibility in the future of immunizing dental personnel through immunostimulation using a vaccine against *L. pneumophila*. This design is characterized by effectiveness, specificity, safety, and stability compared to conventional vaccine development approaches. Using immunoinformatics tools and subtractive proteomics, a vaccine candidate against *L. pneumophila* was designed, which effectively elicited an immune response. However, the final decision regarding its effectiveness will depend on further research [66].

### 9.4. Protective Clothing of Dental Team

The following elements have become and remained the minimum items of dental personal protective equipment (PPE): goggles protecting the eyes, surgical masks, gloves for the hands, as well as protective clothing covering the body, face shields protecting additional areas of the face not covered by masks, and operation caps protecting the hair [67]. The use of personal protective equipment, including face shields and masks, is absolutely essential. Wearing face shields provides a barrier that intercepts splashes and droplets from aerosols, while masks are directly exposed to bioaerosol mist. Face shields protect the facial area and surroundings from splashes and droplets, but not the mask itself. It is important to note that surgical masks are not respiratory protection for the wearer. They protect the patient from exhalation emissions from the wearer and provide some incidental barrier protection only for the wearer.

The recommendation for patients to rinse their mouths with 0.1% chlorhexidine (CHX) primarily reduces microbial contamination of masks. The highest level of microbial contamination on both face shields and masks occurs when patients do not rinse their mouths at all, a lower level is observed when they rinse with water before the procedure, and the lowest contamination is noted when patients rinse with CHX [68]. Considering the routes of infection with *Legionella* bacteria, it is essential to minimize contamination, especially of surgical masks, among members of the dental team. As it turns out, microbial contamination of masks can be reduced by having the patient rinse their mouth with CHX and by wearing a face shield, but it cannot be completely prevented [69].

## 10. Ensuring the Proper Microbiological Quality of the DUWL Environment

To limit microbiological contamination of DUWLs and/or the formation of biofilm, the most optimal approach is the combination of chemical and non-chemical methods. Chemical methods involve the use of various disinfectants according to established protocols, either continuously or through periodic shock treatment. Non-chemical methods, on the other hand, include the use of different water sources supplying the DUWLs and various DUWL flushing protocols, as well as the use of additional dental unit equipment, such as anti-stagnation devices, the installation of antibacterial filters, and other measures, which will be described below.

From the findings of previous studies cited by Tuvo et al., it follows that the continuous use of disinfectants in DUWLs is remarkable in preventing the contamination by *Legionella* and *P. aeruginosa*. The authors state that a promising alternative in combating DUWL colonization by *Legionella* is the installation of water filters, the regular application of 6% *v*/*v* hydrogen peroxide shock disinfection, and the use of surfactants. It is important to emphasize that the aforementioned authors have developed and published detailed guidelines for conducting shock disinfection procedures [37].

One of the non-chemical methods involves the appropriately frequent and sustained flushing of DUWLs. It is recommended to flush the DUWLs in the morning for at least two minutes before starting treatments, and for 20 s before each patient’s treatment. Additionally, to minimize the risk of backflow contamination, flushing should be performed again at the end of the working day [26].

Previously published guidelines by the Organization for Safety, Asepsis, and Prevention (OSAP) recommend that DUWLs be flushed for 20–30 s at the beginning and end of each workday, as well as after each patient, to remove microbiological material potentially backflowed into the DUWL during treatment [65].

It should be emphasized that this flushing should apply to all working ends of the dental unit, including the suction system.

Research conducted in French dental offices shows that flushing, as the primary tool for improving DUWL water quality, was used in 65.4% of offices, chemical disinfection in 62.1%, while water quality analysis was carried out in only 2.6% of the offices [44].

Chemical methods of improving the microbiological quality of water in DUWLs.

In the case of chemical methods, DUWL decontamination is carried out using chemical agents, preferably by combining intermittent and continuous disinfection. Much earlier studies have shown that performing shock disinfection before continuous treatment provides a faster effect in terms of reducing the number of bacteria in the DUWL [70]. Moreover, in daily practice, a combined protocol of continuous and periodic disinfection using different active products was more effective than continuous or periodic disinfection alone in controlling bacterial contamination of water in the DUWL [71,72]. In the U.S. setting, based on survey studies, 56% of dental offices use daily maintenance products, 51% use continuous maintenance products (such as straws or cartridges), 45% perform periodic shock treatments, while 4% do not routinely use any DUWL treatment, and 3% are unaware of which DUWL disinfection methods are being used [49].

According to the study by Baudet et al., chemical water treatment was used in 62.1% of dental units in Eastern France. Earlier studies cited by Baudet and colleagues indicated that in Europe, 45% of dentists reported using water treatment in dental units, while in Eastern England, 50% of dentists did so. The highest rate was recorded in another French department, where 88% of dentists chemically treated water—71% used continuous treatment either alone or combined with point-of-use treatment, and 21% used point-of-use treatment exclusively [44].

The products used for chemical treatment of water in dental care units studied in Eastern France in 2016 were Calbenium^®^ (containing quaternary ammonium, EDTA, and sodium tosylchloramide); Dentosept^®^, Oxygenal^®,^ and XO^®^ water cleaners (based on H_2_O_2_); ICX^®^ (containing sodium percarbonate, silver nitrate, and cationic surfactants); Sterispray^®^ (containing benzalkonium chloride, chloramine T, and EDTA); Alpron^®^/Bilpron^®^ (containing EDTA and polyaminopropyl biguanide with sodium tosylchloramide for Alpron^®^ and with ester p-hydroxybenzoate for Bilpron^®^) [44].

The team of Baudet et al. in other later studies, evaluated the effectiveness of combining an initial shock disinfection aimed at removing potential bacteria and biofilms inside the DUWL, performed using ICX Renew (A-dec, Inc., Newberg, OR, USA), with continuous use of the disinfectant agents Alpron and Bilpron (Alpro Medical GmbH, Bensheim, Germany). This approach allowed for the assessment of their effectiveness. ICX Renew contains hydrogen peroxide, sodium lauryl sulfate, and maleic acid. Alpron, composed mainly of ethylenediaminetetraacetic acid (EDTA), polyhexamethylene biguanide, and sodium tosylchloramide, was used during active periods (Monday to Friday). Bilpron, containing EDTA, polyhexamethylene biguanide, and para-hydroxybenzoate ester, was used during inactivity periods (weekends). The procedure was as follows: on Friday, ICX Renew was flushed out and replaced with Alpron diluted to a 1% concentration in a bottle with sterile water, serving as a disinfectant for continuous maintenance of DUWL water quality. Bilpron, a ready-to-use solution, was used undiluted during inactivity periods exceeding 24 h in the DUWL. The implemented protocol yielded positive results, enabling long-term control of the microbiological quality of water [73].

### 10.1. Chemical Methods of Improving the Microbiological Quality of Water in DUWLs

#### 10.1.1. Hydrogen Peroxide

An effective method for controlling microbiological contamination in DUWLs was the combination of periodic and continuous disinfection using hydrogen peroxide. The periodic disinfection involved filling the waterlines with a disinfectant solution (1.4% hydrogen peroxide) for 24 h, while the continuous disinfection consisted of treating the water (0.014% hydrogen peroxide) used for dental procedures and flushing the waterlines with it for 2 min each morning before procedures, as well as flushing the waterlines for 30 s after each dental procedure. This contributed to the loosening of the biofilm structure and the reduction in a large number of biofilm-forming bacteria [74].

#### 10.1.2. Chlorine Dioxide (ClO_2_)

Chlorine dioxide (ClO_2_) is a strong, selective oxidizer and a stable agent over a wide pH range. According to studies by Krüger et al., ClO_2_ is an effective disinfectant against microorganisms found in the water systems of dental offices. It was shown that Gram-negative bacteria are more susceptible than Gram-positive bacteria to the action of this disinfectant, and ClO_2_ is effective in disinfecting waterborne microorganisms in their planktonic state [75].

Also, after thorough research, Yue C. et al. recommend the use of a chlorine dioxide (ClO_2_)-based disinfectant at a concentration of 20 mg/L to significantly reduce bacterial biofilm in the water lines of dental unit waterlines (DUWLs). In their study model, the disinfectant was used to treat biofilms of *S. aureus* and *E. coli*. It is important to note that this concentration also provides satisfactory cellular safety and metal corrosion resistance. With its low toxicity and mild corrosiveness, ClO_2_ holds potential for enhancing safety and reducing infection risks [76].

#### 10.1.3. Chlorine Dioxide (ClO_2_) and Hypochlorous Acid (HOCl)

The effect of chlorine dioxide (ClO_2_) and hypochlorous acid (HOCl) on microorganisms and biofilm in DUWLs was investigated, and it was shown that bacterial growth in the devices was inhibited within 5–10 min using both disinfectants. The difference in biofilm formation before and after the application of each disinfectant was significant, as confirmed by SEM (Scanning Electron Microscopy) examination [63].

#### 10.1.4. Chlorine and/or Hydrogen Peroxide

German studies found no significant differences between two disinfection procedures in terms of *Legionella* contamination (Kruskal–Wallis test; H(2) = 0.573, *p* = 0.751). The disinfection system was based on the use of oxidizing agents such as chlorine and/or hydrogen peroxide. With regular disinfection, the operating water in dental units was mixed with hydrogen peroxide, and chlorine-based products were often additionally added to the water supplied to the dental chair, for example via bottle systems [26].

#### 10.1.5. Chlorogenic Acid Solution

Attention has been drawn to the effectiveness of using a chlorogenic acid solution at a concentration of 0.025 g/mL over a three-month period in monitoring microbiological contamination in DUWL. The disinfection influenced the microbiological profile–reducing the pathogenicity of the bacterial community in the DUWL, while also promoting the growth of various probiotic strains. Chlorogenic acid showed significant inhibitory properties against opportunistic pathogens such as Mycobacterium, while also promoting the growth of diverse probiotic strains, as confirmed by pyrosequencing analysis. According to the author, it would be interesting to conduct studies on the effectiveness of this method against *L. pneumphila* [77].

#### 10.1.6. Silver Nanoparticles

Silver nanoparticles (AgNPs) possess a broad spectrum of antibacterial, antifungal, and antiviral properties. They have the ability to penetrate bacterial cell walls, altering membrane structures, and can even lead to bacterial cell death. Their effectiveness is attributed not only to their nanoscale size but also to their high surface-to-volume ratio. Silver nanoparticles can increase membrane permeability, generate reactive oxygen species (ROS), and interrupt the replication of deoxyribonucleic acid (DNA) through the release of silver ions [78].

#### 10.1.7. Nanosilver and Hydrogen Peroxide Molecules

The disinfectant efficacy against *L. pneumophila* of an and Ag^+^-based disinfectant (H_2_O_2_ 3% *v*/*v*, Ag^+^ 0.001% *w*/*v*) had already been confirmed much earlier. After just 10 min, it achieved a 5.4 mean log CFU load reduction for *L. pneumophila*. It was recognized that this type of disinfection could help minimize the risk that planktonic pathogens are spread to patients during dental treatment [79].

Hydrogen peroxide (Peroxy Ag^+^), a disinfectant based on 3% H_2_O_2_ with 0.001% Ag^+^, produced by Cefla S.C., Imola, Italy, proved effective against *Legionella* bacteria; it obtained an average reduction of 85% of *Legionella* load. The product is effective in reducing the number of Legionella cells after 75 min of contact time (99.997%) [61].

Hoogenkamp et al. found out that units which are administered hydrogen peroxide-based products as a daily low dose disinfectant contained a significantly lower HPC, bacterial 16S rDNA and *Legionella* spp. DNA concentration and administering an additional weekly shock dose of hydrogen peroxide, silver ions, or hypochlorous acid appeared to further prevent bacterial growth. The mechanism of hypochlorous acid, a powerful disinfectant, was utilized—it acts by disrupting the integrity of the cell membrane—along with silver ions, known for their interaction with the bacterial membrane and binding to phosphate groups during DNA replication, to inhibit the replication of *Legionella* at relatively low concentrations [28].

A systematic review of the literature by Hong et al., which aimed to examine the evidence supporting the efficacy and safety of silver use for DUWL decontamination, concluded that there is good evidence of antimicrobial efficacy of silver with hydrogen peroxide on diverse microorganisms present in DUWLs. Furthermore, there is insufficient evidence on the application of silver nanoparticles (AgNPs) as an efficient material to control the biofilms in DUWLs. There is evidence suggesting that continuous monitoring using H_2_O_2_/silver is likely an effective method for combating biofilm present on the inner walls of waterlines. However, such properties were not confirmed when evaluating commercial silver-containing disinfectant tablets, such as ICX© and Citrisil©, which demonstrated antimicrobial effectiveness against heterotrophic bacteria in dental unit water but failed to control biofilm composed of bacteria originating from dental water sources [80].

From the health risk perspective related to *Legionella* bacteria present in DUWLs, it is crucial to focus on the eradication of environmental free-living amoebae (FLA), such as *Hartmannella*, which act as vectors for *Legionella*, and should therefore be treated as indirect human pathogens. Thus, it is essential to ensure that disinfectants are effective against both amoebae and *Legionella* cultivated inside them, in order to reduce water contamination in DUWLs [81]. Among the common disinfectants historically used in DUWLs (chlorine, hydrogen peroxide (H_2_O_2_), and Oxygenal 6©), none of these tested disinfectants were able to eradicate *Hartmannella vermiformis*. Oxygenal 6© proved to be the most effective in preventing the growth of three species of Candida; chlorine was effective but only at highest doses tested, and H_2_O_2_ had no significant activity [82].

### 10.2. Other Methods of Improving the Microbiological Quality of Water in DUWLs

1. Assuming that waterlines (DUWLs) in newly installed dental units may be microbiologically contaminated, an initial shock disinfection should be carried out before the first clinical use. As demonstrated, this approach ensures a faster reduction in bacterial load in the DUWL compared to the use of continuous disinfection alone. This is crucial as a preventive measure against infections [83,84].

2. The installation of an antiretraction adapter (ARA) attached to the handpiece (HP) could contribute to improving the quality of water in DUWLs. The main idea behind it is to prevent the backflow of fluids from the oral cavity into the DUWL. Studies show that this device has an impact on reducing the total number of bacteria in the water flowing from the unit’s handpieces compared to handpieces without the ARA. It should be noted that the results were not statistically significant. This was confirmed using PCR, Legionella-specific PCR, and culture-based analysis. Additionally, no difference was observed in the bacterial load of water taken from both the handpiece (HP) and the DUWL. Therefore, it was concluded that the ARA was ineffective in reducing the *Legionella* species load in the DUWL. However, considering that the ARA device is commercially available, inexpensive, and easy to install, and the observed lower bacterial load in water flowing from handpieces with ARA compared to those without, it is worth considering the installation of the device [85].

3. Knowledge about the causes and mechanisms of biofilm formation in DUWLs serves as a turning point in research on so-called smart coatings for water line walls. These coatings are elements that can prevent the proliferation of microorganisms, including *Legionella*, in DUWLs. The coatings of tubes forming DUWLs can hinder biofilm formation by preventing bacteria adhesion, creating a spherical, mechanical, and/or electrostatic barrier. Other coatings exhibit bactericidal properties against microorganisms before or after contact with the surface layer. Sciuto et al. proposed a new smart surface against L. pneumophila biofilm formation. This is based on an innovative type of coating consisting of a sulfonated pentablock copolymer (s-PBC, commercially named Nexar™) deposited on top of a polypropylene (PP) coupon in a sandwich filter model. The covering of PP with s-PBC results in a more hydrophilic, acid, and negatively charged surface that induces microbial physiological inhibition thereby preventing adhesion and/or proliferation attempts of *L. pneumophila* prior to the biofilm formation [86].

4. Research is also ongoing on coatings for DUWL tube walls with durable and renewable antibacterial activity. The work by Xing et al. presents a new approach to designing durable and renewable antibacterial dental unit waterlines to prevent and reduce the frequency of hospital-acquired infections. A polymer coating of N-halamine has been developed for dental unit waterlines, which possesses durable and renewable antibacterial activity through the repeatable regeneration of active chlorine. The N-halamine polymer coating exhibits a hydrophilic surface by forming a hydration layer through electrostatic interactions and hydrogen bonding, which can inhibit biofouling deposition and block the halide active site on waterlines, thus helping to maintain the cleanliness of the coating. Additionally, the N-halamine polymer coating exhibits significant antibacterial activity against *E. coli* and *S. aureus*, effectively inhibiting the formation of bacterial biofilms. This is primarily due to the high positive charge density on the surface of the coating and the release of active chlorine. Furthermore, the coating demonstrated good biocompatibility. It is recommended to continue research focused on evaluating the effectiveness of this antibacterial coating against *Legionella* spp. [87].

## 11. Summary

In light of the current data cited, a comprehensive strategy for preventing cross-infection, including *L. pneumophila*, in a dental office should take into account several important factors (aspects). These include the microbiological quality of water, the presence of biofilm in the DUWL, detection methods for the presence of *Legionella*, chemical and non-chemical methods ensuring proper microbiological quality of the DUWL and dental office environment, and the principles of patient and staff protection.

## Data Availability

The data presented in this study are available on request from the corresponding author. The data are not publicly available due to privacy.

## Abbreviations

The following abbreviations are used in this manuscript:

16S rRNA	RNA component of the 30S subunit of a prokaryotic ribosome
AC	Air Cleaner
ADA	American Dental Association
AgNPs	Silver Nanoparticles
ARA	Anti Retraction Adapter
C-100	the European Union standard, whose criterion is more than 100 CFU/mL
CDA	Cultivation-Dependent Analysis
CDC	American Centers for Disease Control and Prevention
C-di-GMP	Cyclic Diguanylate
CFU	Colony-Forming Unit
CFU/L	colony-Forming Unit in Liter
CFU/mL	Colony-Forming Unit in Mililiter
CHX	Chlohexidine
CIA	Cultivation-Independent Analysis
ClO_2_	Chlorine Dioxide
DLDD	Daily Low Dose Disinfectant
DSM	Dip Slide Technique mMethod
DUWL(s)	Dental Unit Waterline(s)
*E. coli*	*Escherichia coli*
EDTA	Ethylenediaminetetraacetic Acid
FCM	Flow Cytometry
FLA	Free-Living Amoebae
H_2_O_2_	Hydrogen Peroxide
HOCL	Hypochlorous Acid
HPC	Heterotrophic Plate Count
HVE	High-Volume Evacuation system
LAMP	Loop-mediated Isothermal Amplification
*L. anisa*	*Legionella anisa*
*L. pneumophila*	*Legionella pneumophila*
*L. rubrilucens*	*Legionella rubrilucens*
*Legionella* spp.	several unidentified or not yet described species within a genu of *Legionella*
LVE	Low-volume Evacuation system
MALDI–TOF MS	Mass Spectrometry
MeSH	Medical Subject Headings
NTM	*Nontuberculous mycobacteria*
OSAP	Organization for Safety, Asepsis, and Prevention
*P. aeruginosa*	*Pseudomonas aeruginosa*
PCR	Polymerase Chain Reaction
PP	Polypropylene
*Pseudomonas* spp.	Several unidentified or not yet described species within a genu of *Pseudomonas*
R2A agar	A culture medium for bacteria from potable water or other environments
Rpm	Revolutions per minute
*S. aureus*	*Staphylococvuss aureus*
SEM	Scanning Electron Microscopy
S-PBC	Sulfonated Pentablock Copolymer
SSM	Surface Smear Method
UE	European Union
UV	Ultraviolet
WHO	World Health Organization
